# Silencing of miRNA-148a by hypermethylation activates the integrin-mediated signaling pathway in nasopharyngeal carcinoma

**DOI:** 10.18632/oncotarget.2282

**Published:** 2014-07-31

**Authors:** Hsin-Pai Li, Hsin-Yi Huang, Yi-Ru Lai, Jing-Xuan Huang, Kai-Ping Chang, Chuen Hsueh, Yu-Sun Chang

**Affiliations:** ^1^ Graduate Institute of Biomedical Sciences, Chang Gung University, Taoyuan, Taiwan, Republic of China (ROC); ^2^ Molecular Medicine Research Center, Chang Gung University, Taoyuan, Taiwan, Republic of China (ROC); ^3^ Department of Microbiology and Immunology, School of Medicine, Chang Gung University, Taoyuan, Taiwan, Republic of China (ROC); ^4^ Pathology Core, Chang Gung University, Taoyuan, Taiwan, Republic of China (ROC); ^5^ Department of Pathology, Chang Gung Memorial Hospital at Lin-Kou, Taoyuan, Taiwan, ROC; ^6^ Otolaryngology-Head and Neck Surgery, Chang Gung Memorial Hospital at Lin-Kou, Taoyuan, Taiwan, ROC

**Keywords:** miR-148a, DNA methylation, migration, nasopharyngeal carcinoma, integrin-signaling pathway

## Abstract

MicroRNAs (miRNAs) play a pivotal role in carcinogenesis by suppressing oncogenes or tumor suppressor genes. Various studies have identified numerous miRNAs and their diverse targets; however, the consequences of dysregulated miRNAs in nasopharyngeal carcinoma (NPC) remain unclear. For this study, we found that miR-148a is downregulated through hypermethylation in NPC biopsies and NPC cell lines compared with adjacent normal and NP cells respectively. Promoter assays demonstrated that upstream stimulatory factor 1 (USF1) is a crucial transcription factor that activates miR-148a promoter activity. EMSA assays confirmed that purified USF1 binds better toward the unmethylated than the methylated CG-containing USF1 consensus probe. The ectopic expression of miR-148a inhibits cell migration in NPC cells through the suppression of integrin-mediated signaling by targeting VAV2, WASL and ROCK1. Biochemical and functional assays provided supporting evidence that these 3 genes are the downstream targets of miR-148a in NPC cells. Furthermore, immunohistochemical staining and Western blotting analysis revealed that the 3 oncogenic targets of miR-148a were overexpressed in NPC biopsies, suggesting that the inactivation of miR-148a caused by DNA methylation promotes NPC progression. Overall, our findings revealed that miR-148a can act as tumor suppressor miRNA and serve as a biomarker as well as a therapeutic target for NPC.

## INTRODUCTION

Nasopharyngeal carcinoma (NPC) is a highly invasive head and neck cancer associated with EB virus infection [1]. The EBV latent membrane protein 1 (LMP1) is the major transforming viral protein that contributes to NPC development [2-4]. It has been demonstrated that LMP1 up-regulates DNA methyltransferases (DNMTs) in NPC and causes the inactivation of tumor suppressor genes (TSGs) such as E-cadherin [5], RASSF1A [6 ], p16 [7] and HoxA2 [8] through hypermethylation. DNA methylation-mediated gene silencing not only affects coding genes but also non-coding genes such as microRNAs (miRNAs) [9].

The miRNAs are endogenous short RNAs (approximately 22-nt) that downregulate gene expression by partially or completely complementing the 3’-untranslated region (UTR) of target genes. Based on the computational prediction, TargetScan, each miRNA may have 500 to 800 potential targets [10]. Numerous studies have shown that miRNAs play a crucial role in cellular progression such as proliferation, metastasis, apoptosis, and development by suppressing the expression levels of its target genes [11]. The dysregulation of oncogenes and TSGs promotes tumor development has been well documented. Although miRNAs are non-coding genes, miRNA dysregulation also contributes to tumor progression and formation [11-14]. For understanding the miRNA expression profile in cancers, miRNA microarray is a widely used technique to screen differentially expressed miRNAs in cancers. To identify miRNAs that are dysregulated because of aberrant DNA methylation in NPC, we performed miRNA microarray to compare miRNA expression profiles in normal nasopharynx (NP) and NPC cells with or without treatment with the DNA demethylation agent 5-aza-2’deoxycytidine (5’aza). Our miRNA microarray data showed that miR-148a is downregulated and hypermethylated in NPC. Previous studies have shown that miR-148a is downregulated in various of cancers such as hepatocarcinoma [15]; pancreatic ductal adenocarcinoma [16]; and gastric [17], ovarian [18], breast [19], non-small cell lung [20], and colorectal cancers [21]. According to these studies, miR-148a suppresses cell growth, promotes apoptosis, and inhibits migration and invasion, indicating that it plays a tumor-suppressive role in normal tissues.

In this study, we demonstrated that DNA methylation downregulates miRNA-148a in NPC. Further investigations revealed that miR-148a represses cell migration by targeting 3 genes involved in the integrin pathway, including guanine nucleotide exchange factor VAV2, Wiscott-Aldrich syndrome-like protein (WASL), and Rho kinase 1 (ROCK1). Finally, our data showed that miR-148a represses the integrin-mediated signaling pathway, and inhibits oncogenic targets that engage in cell migration. Conversely, the inactivation of miR-148a through aberrant hypermethylation promotes NPC tumorigenesis.

## RESULTS

### miR-148a is downregulated in both primary NPC tissues and cell lines

To identify the miRNAs that are repressed because of methylation in NPC, we collected RNA from EBV-containing NPC cell line C666.1 treated with or without 5’aza, which we then converted to cDNAs, before conducting miRNA microarray analysis to monitor the miRNA expression profile (Agilent; human miRNA Oligo microarray R12). According to the microarray data, we identified 6 of 866 human miRNA genes with a miRNA expression that could be at least 1.5-fold restored after the addition of 5’aza (Supplementary Table 1). The preliminary results indicated that miR-148a might be downregulated because of DNA methylation in NPC. A previous report showed that the expression of miR-148a was also downregulated by approximately 6-fold in NPC biopsies compared with adjacent normal tissues [12], supporting our observation in NPC cell lines.

**Figure 1:**
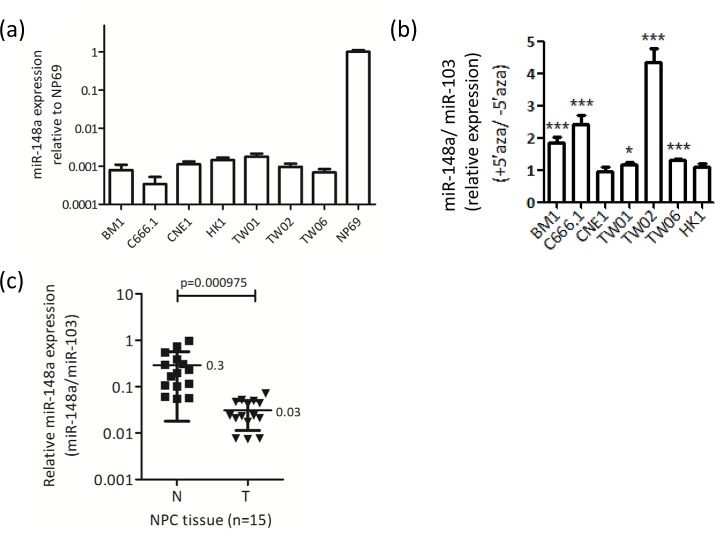
miR-148a is downregulated by DNA methylation in NPC cell lines and biopsies (a) qRT-PCR analysis of miR-148a expression was performed on 7 NPC cell lines and NP69. The results were normalized to miR-103. (b) The columns represent the relative fold change of miR-148a expression in NPC cell lines treated with or without 5’aza (10μM). (c) The expression level of miR-148a in 15 paired NPC biopsies were detected by qRT-PCR. Data represent the relative fold change of miR-148a expression in the log_10_ value to that of miR-103 (N: adjacent normal tissues; T: NPC tumors).

To confirm that the *miR-148a* gene is hypermethylated in other NPC cell lines, we analyzed the miR-148a expression level by conducting stem-loop qRT-PCR in 6 NPC cell lines with or without treatment of the DNA methylation inhibitor 5’aza. In the presence of 5’aza, miR-148a expression increased by approximately 2- to 4-fold in BM1, C666.1, and TW02 cell lines (Fig. 1a), suggesting that DNA methylation regulates *miR-148a* in NPC cells. We also analyzed the expression levels of miR-148a in NP69, a relatively normal NP cell line, immortalized through large T-antigen [22], and NPC cell lines. The results showed that the expression levels of miR-148a were significantly down-regulated (approximately 1000-fold) in NPC cell lines compared with NP69 cells (Fig. 1b). We further analyzed the expression levels of miR-148a in 15 pairs of NPC biopsies. Consistent with the results obtained in NPC cells, miR-148a expression levels were suppressed by 2- to 52-fold (mean 10-fold) in NPC tumors compared with corresponding non-tumors. The miR-148a expression (normalized with miR-103) in NPC tissues was low (mean = 0.03, 10-fold lower) compared with that in adjacent normal tissues (mean = 0.3) (Fig. 1c). Overall, these data revealed that miR-148a expression is suppressed in both NPC cell lines and tumors, and that the reduction of miRNA expression is likely due to DNA hypermethylation.

### Identification of differential methylation region (DMR) of miR-148a

**Figure 2:**
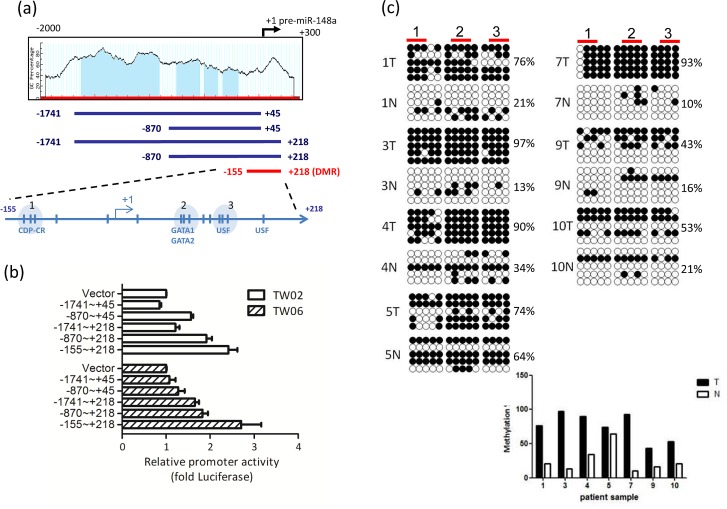

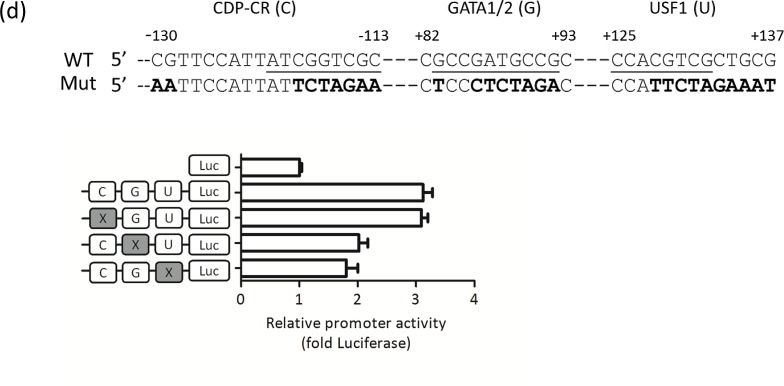

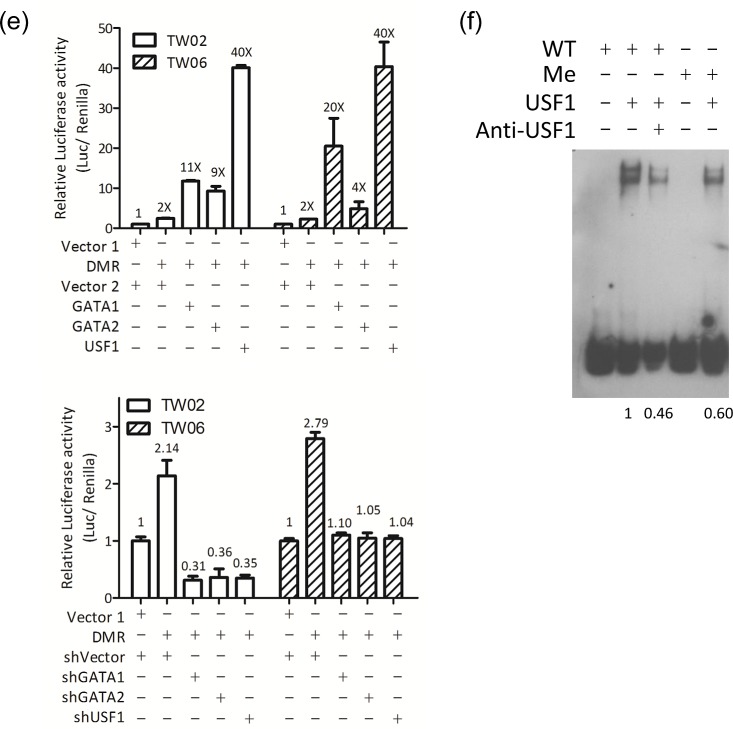
miR-148a is hypermethylated in NPC (a) Schematic map containing CpG islands (shaded area) of the miR-148a upstream 2Kb region, as adapted from the MethPrimer website (upper panel), indicating the transcription start site (+1), 15 CpG sites (vertical bars), and the transcription factors (CDP-CR, GATA1, GATA2, and USF1) overlapping the CpG sites. 5 miR-148a promoter deletion clones were indicated (lower panel). (b) Different miR-148a promoter deletion clones were transfected into TW02 and TW06 cells. Relative luciferase activities were measured 48 h post-transfection, and all the luciferase reporter activities were normalized to that of the renilla activities. (c) Bisulfite sequencing analysis was performed on miR-148a (-155 to +186) in 7 paired NPC clinical samples. Each horizontal row represents a single clone; the methylation percentages of 5 individual clones were indicated. The white and black circles represent the unmethylated and methylated CpG sites, respectively. The methylation percentage of each sample was shown in lower panel. (d) Different mutated miR-148a DMR clones were transfected into TW02 cells. Site direct mutagenesis was performed, and the primers were shown in Supplementary Table 5. Bold letters represent mutated sequences, and underlined texts represent the consensus sequences of the transcription factor. (e) Luciferase reporter containing miR-148a DMR and the transcription factor expressing clones (GATA1, GATA2, and USF1) (upper panel) or shGATA1, shGATA2, and shUSF1 (lower panel) were cotransfected as indicated into TW02 and TW06 cells lines. Cells were harvested and luciferase assays were performed after 48 h. (f) EMSA assay was performed by using a 50 fmole wild-type (WT) biotinylated probe (mir-148a promoter containing USF1 binding sequence), or a methylated probe (Me); and the purified bacteria-recombinant USF1 protein (5μg). Anti-USF1 antibody (2μg) was used in super-shift experiments. The relative intensity of USF1 binding was indicated.

To identify the possible differentially methylated CpG sites of *miR-148a*, we subjected the 2Kb upstream DNA sequence of pre-miR-148a to (1) web-based prediction algorithms by MethPrimer to predict the potential CpG island; and (2) TFSearch to predict the transcription factor binding sites (Fig. 2a). We identified 4 putative CpG islands (-1627 to -912; -753 to -540; -501 to -377; and -333 to -192) as shown in Fig. 2a. We then performed bisulfite sequencing to confirm whether these CpG islands were differentially hypermethylated in the control and 5’aza-treated C666.1 cells. However, we noted no significant difference in the methylation percentage of each CpG island (approximately 60%) between the control and treated C666.1 cells (data not shown), suggesting that the differential methylated region (DMR) may not be situated in the 4 predicted CpG islands. A recent report showed that the DMR of *miR-148a* is located at -155 to +186, which is outside the predicted CpG islands in pancreatic cancer [23]. We then further confirmed the methylation status of the *miR-148a* region with bisulfite sequencing in 7 of 15 paired NPC tissues. The results showed that the CpG sites within -155 to +186 of miR-148a in 7 NPC tumor tissues were differentially hypermethylated (53% to 97%, mean 72.6%) compared with that of their adjacent normal tissues (10% to 64%, mean 25.6%) (Fig. 2c) indicated that the methylation status of NPC tumors was high compared with that of the non-tumors.

To determine whether the DMR region overlaps with the minimal regulatory sequences that govern miR-148a transcription, we generated 5 miR-148a upstream deletion luciferase reporter constructs, as shown in Fig 2a, and performed promoter activity assays in NPC cells. We confirmed that -155 to +218 (15 CpG sites) is the minimal region for *miR-148a* promoter activity in TW02 and TW06 cell lines (Fig. 2b). Transcription factor binding site prediction focusing on -155 to +186 showed that 3 major CG-containing transcription factor binding sites: CDP-CR, GATA1/2, and USF1, within the DMR (Fig. 2a, lower panel). To identify which of these sites are crucial for promoter activation, we generated 3 “site-directed mutant reporters” (-130 to -113; +82 to +93; and +125 to +137), and each mutant contained one mutated site (Fig. 2d, upper panel) to disrupt transcription factor binding. Luciferase assays revealed that the promoter activity of the CDP-CR mutant reporter was the same as that of the wild type (WT), but the GATA1/2 and USF1 mutant reporters were approximately 30% less than that of the WT DMR (Fig. 2d, lower panel). These data indicated that GATA1/2 and USF1, but not CDP-CR, are crucial sites for miR-148a promoter activity. The co-transfection of one of the expression clones GATA1, GATA2, or USF1, with the DMR promoter reporter significantly activated DMR promoter activities by 9- to 40-fold in the TW02 cells, and 4- to 40-fold in the TW06 cell (Fig. 2e, upper panel). The overexpression of USF1 had the greatest impact on promoter activation. To test which transcription factors had the most significant effect on DMR activity, we subsequently attempted to knock down the expression of the endogenous transcription factors GATA1, GATA2, and USF1 by using plasmids expressing individual shRNA, respectively. All 3 individual shRNAs substantially reduced the DMR promoter activities to similar levels, by approximately 6-fold in TW02, and by approximately 2-folds in TW06 cells, compared with that of the shVector control (Fig.2e, lower panel). Overall, these results indicated that GATA1, GATA2, and USF1 are involved in DMR activation; however, USF1 is the strongest transactivator among these transcription factors.

Next, we performed EMSA experiments by using probes containing the USF1 consensus binding site that originated from the miR-148a (+125 to +137) DNA sequence, with either the unmethylated CG site or the methylated CG site (*methylcytosine, CA*CGT*CG), to test whether DNA methylation could affect the binding affinity of purified USF1. The purified USF1 bound well to the unmethylated DNA probe; however, the binding affinity between USF1 and the methylated probe appeared to drop by approximately 40%, demonstrating that the 2 methylcytosines interfered with the binding of transcription activator USF1 (Fig. 2f). Therefore, the decrease in USF1 binding to the methylated DNA may correlate with the reduction of the miR-148a transcriptional level in NPC cells.

### miR-148a inhibited cell migration in NPC cells

**Figure 3:**
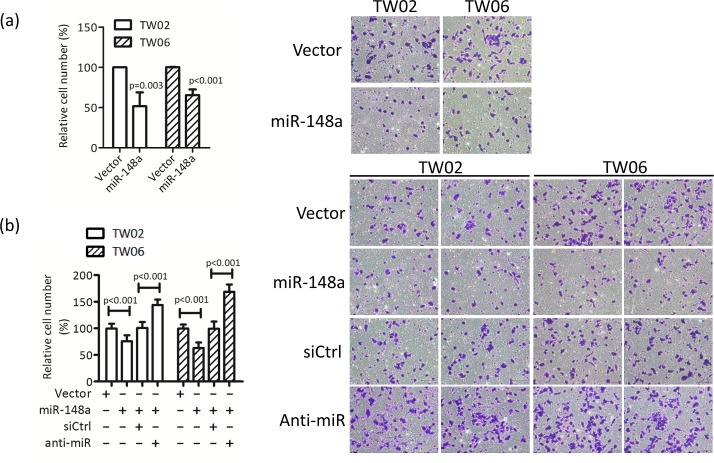

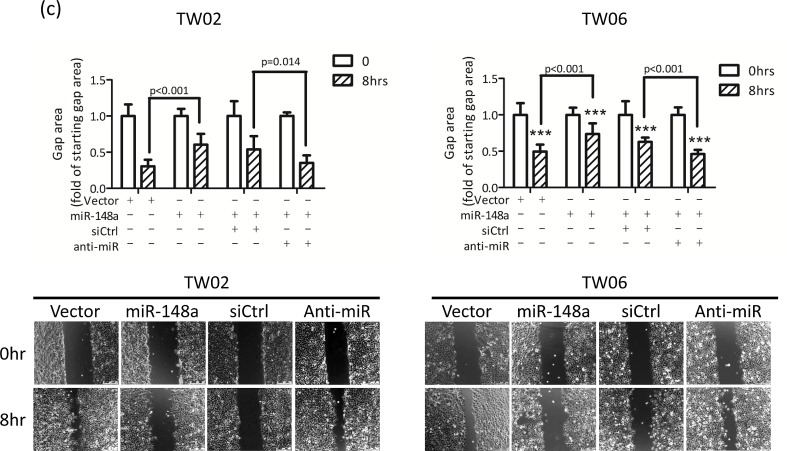
miR-148a inhibits cell migration *in vitro* TW02 and TW06 cells transiently expressing (a) vector control, or miR-148a (1μg); (b) vector control, or miR-148a (1μg); and control (siCtrl) or miR-148a inhibitor (anti-miR, 50 pmole), were used to perform transwell cell migration assay. Migrated cells of 10 microscopic fields from 3 independent, duplicate experiments were counted. (c) Wound-healing assays were performed using similar conditions as in (b). The gap area (wound) was measured and analyzed by ImageJ at the indicated time.

To explore the biological functions of miR-148a in NPC cells, we performed cell proliferation, cell migration, cell invasion, and colony formation assays in the control and in miR-148a overexpressing TW02 and TW06 cells. However, we detected a significant difference only in cell migration assay. Transwell migration assay revealed that overexpressing miR-148a significantly inhibited cell migration in TW02 cells (48%) and in TW06 cells (36%) (Fig. 3a), whereas anti-miR148a promoted cell migration significantly in TW02 cells (43%) and in TW06 cells (68%) (Fig.3b). Furthermore, wound-healing assay showed that overexpressing miR-148a inhibited cell migration in TW02 and TW06 cells by 50% and 33%, respectively, compared with that of the vector control (Fig. 3c). These data demonstrated that miR-148a overexpression can reduce cell migration ability in NPC cells.

### Integrin signaling genes are potential miR-148a targets in NPC

**Figure 4:**
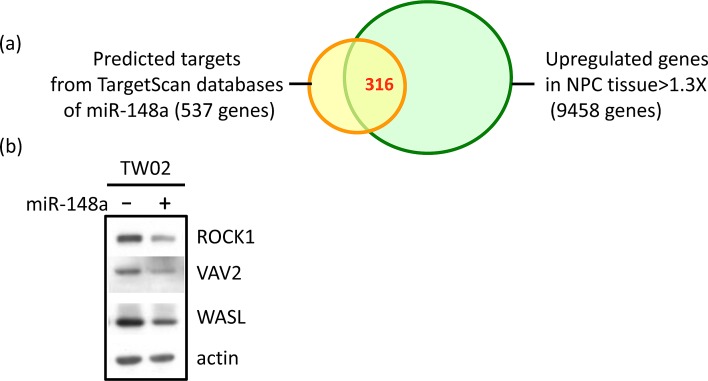

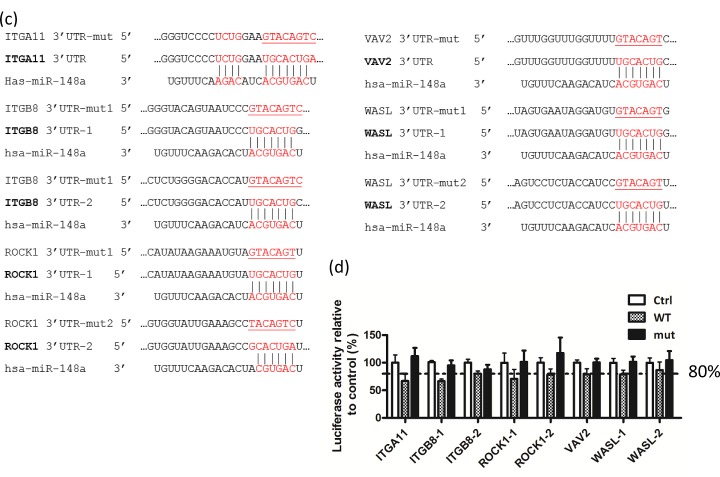
Identification of the target genes of miR-148a and the effect of miR-148a on the targets (a) Partek analysis of upregulated genes in NPC tissues according to microarray data and miR-148a target genes (TargetScan). (b) Western blotting analysis was performed to examine the expression of target genes in miR-148a transient transfected TW02 cells. (c) Base pairing between miR-148a and the putative target site in the 3′UTR of the 5 target genes and the 3′UTR mutant sequences (underlined) were indicated, respectively. The 3′UTR of ITGA11 and VAV2, each has a single miR-148a binding site, whereas the 3′UTR of ITGB8, ROCK1, and WASL, each has two miR-148a binding sites. (d) Luciferase reporters containing WT or mutant 3′UTR binding sites of miR-148a target genes, and vector or miR-148a were co-transfected separately into 293T cells. Luciferase reporter assays were conducted 48 h post transfection. A dotted line represents 80% of the luciferase activity.

The inactivation of miR-148a expression caused by DNA methylation may result in the overexpression of miR-148a target genes. Some of these targets may promote the oncogenic potential of NPC. To identify the oncogenic target genes of miR-148a that were overexpressed in NPC because of miR-148a inactivation, we intersected and analyzed 2 gene lists: (1) the 537 predicted target genes of miR148a from TargetScan (5.2 version) [24], and (2) the 1.3-fold upregulated 9458 genes in 9 NPC tumors when compared with the combined adjacent non-tumors based on the analysis of Affymetrix (u133 plus 2.0) cDNA microarray data, using Partek analysis software. The selected 316 intersected genes were both miR-148a target genes and overexpressed in NPC (Fig. 4a). We further subjected these genes to the Pathway Maps analysis by using MetaCore and selected one of the most significant pathways (Supplementary Table 2). We selected potential miR-148a cancer-related target genes, namely ITGA11, ITGB8, VAV2, ROCK1 and WASL, which are known to be involved in integrin pathway-promoting cell migration, motility, actin polymerization, and cytoskeleton remodeling (Supplementary Table 3).

To further determine whether the potential cancer-related genes are the direct targets of miRNA, we overexpressed miR-148a in TW02 cells and performed qRT-PCR to assess the mRNA expression level of the miR-148a potential targets (Supplementary Table 4). The mRNA expression levels of 4 target genes were downregulated (15%-24%) in miR-148a-overexpressing cells, except the VAV2 gene (Supplementary Table 4). Conversely, miR-148a overexpression repressed VAV2, ROCK1, and WASL protein expression (Fig. 4b), suggesting that miR-148a expression may affect both the mRNA and protein levels of ROCK1 and WASL, whereas it may affect only the protein level of VAV2.

The miRNAs usually target the 3′UTR of the target mRNA through sequence complementarity. The most critical base-pairing nucleotides are the second to eighth bases on the miRNA, also known as “seed sequences” [25]. To determine whether miR-148a could directly target the 3′UTR region of these 5 predicted targets, we constructed luciferase reporters containing the WT or mutant (mut) miR-148a binding sequence found in the 3′UTR of 5 target gene mRNAs (Fig. 4c). The transient co-transfection of miR-148a with the WT miR-reporters in 293T cells exhibited a significant reduction (13% to 33%) in luciferase activity compared with the vector control. However, miR-148a co-transfection with mutant miR-reporters has no effect on luciferase activity, as shown in Fig. 4d. The results demonstrated that miR-148a could specifically target the 3′-UTR of ITGA11, ITGB8, ROCK1, VAV2, and WASL regions by binding to the corresponding miR-148a recognition sequences, and subsequently blocking translation. Overall, these results indicated that miR-148a may specifically target these 5 genes in the integrin signaling pathway, and suppress the protein expression of ROCK1, VAV2, and WASL.

### MiR-148a inhibits migration through targeting integrin signaling genes

**Figure 5:**
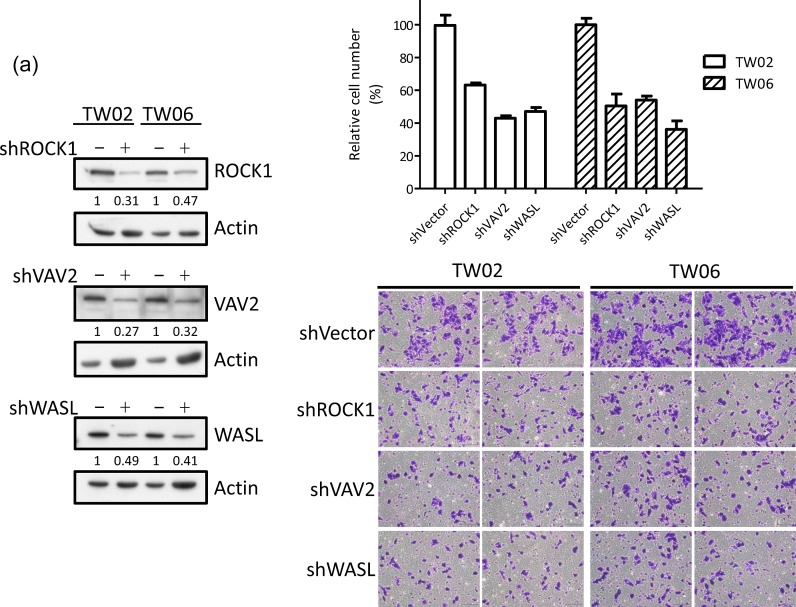

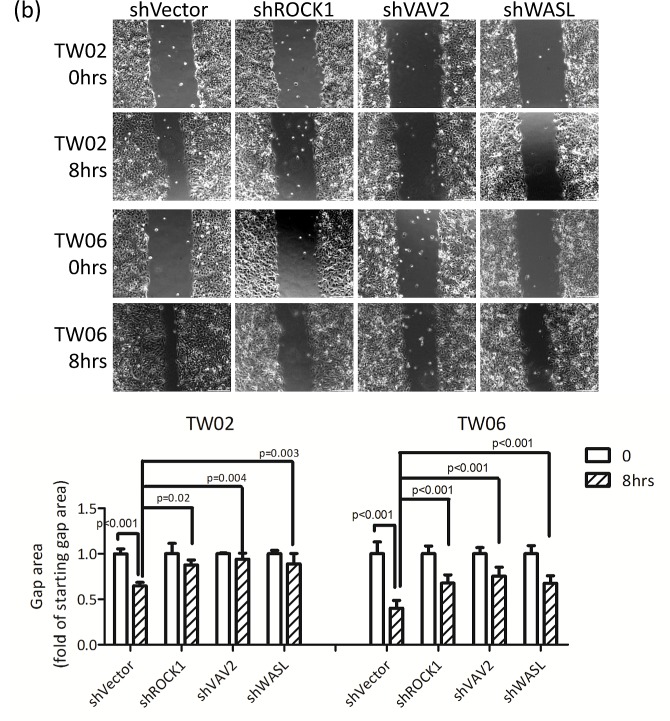

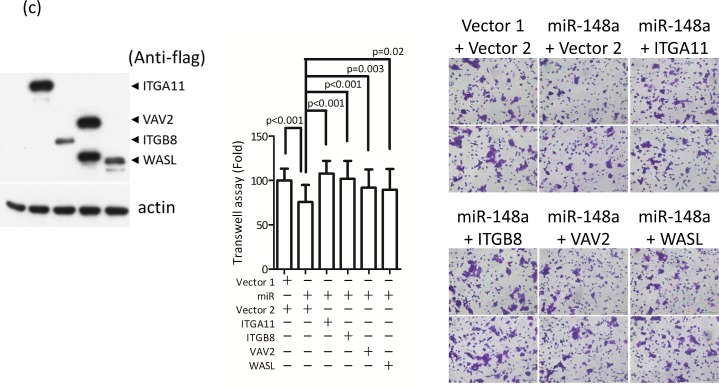

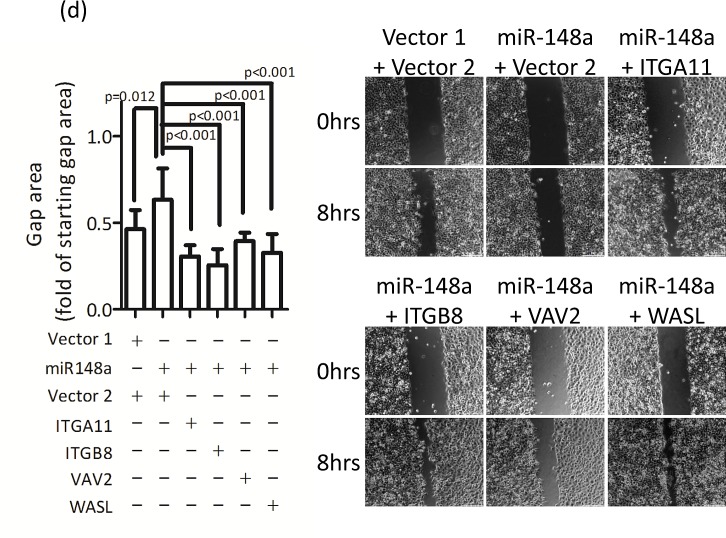

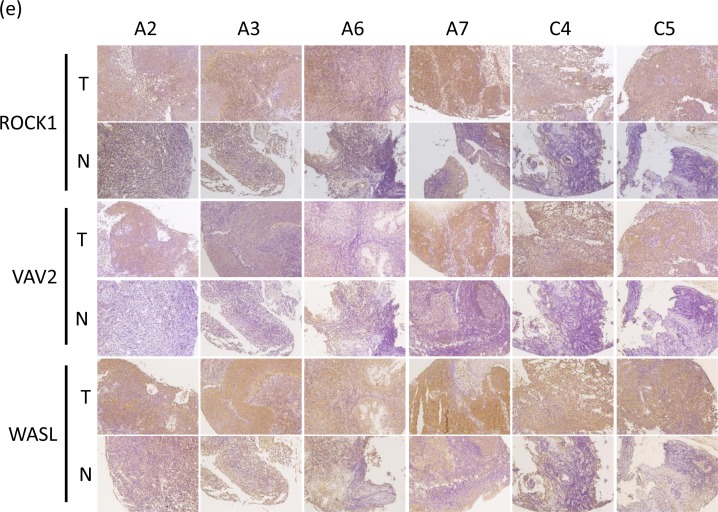

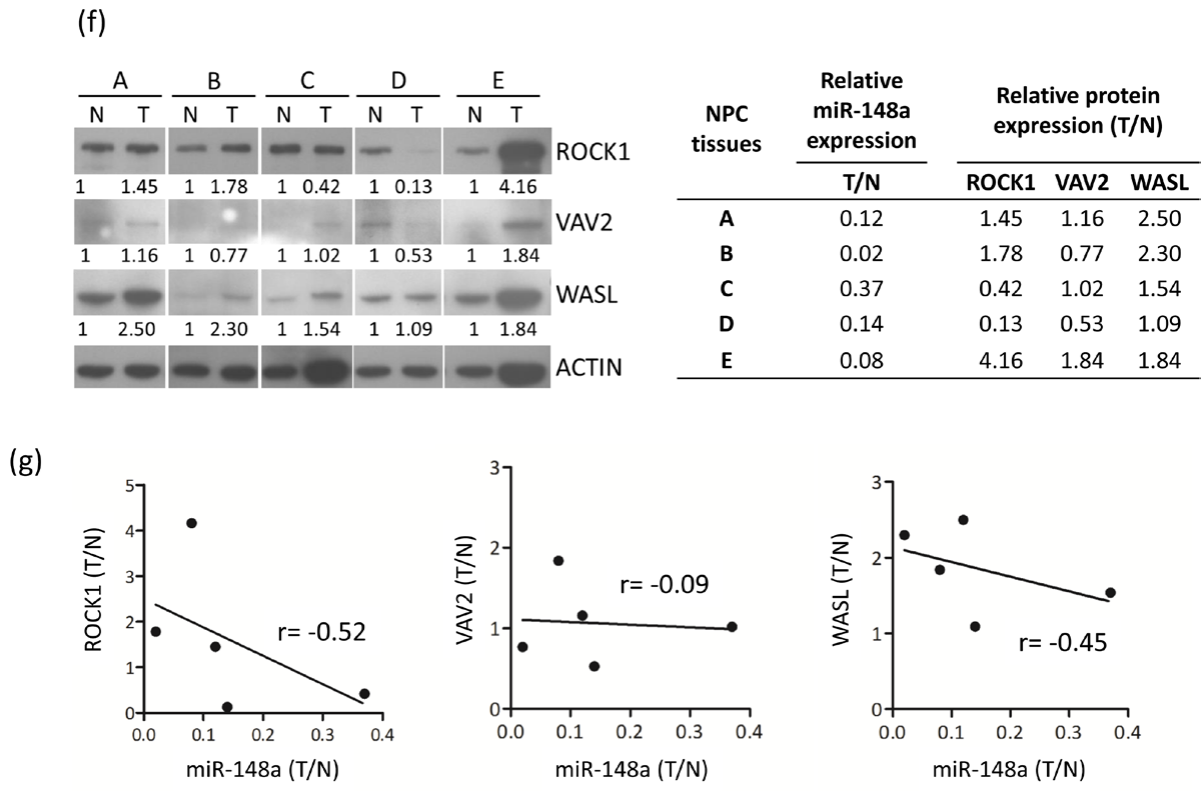
miR-148a inhibits integrin signaling pathway by targeting ITGA11, ITGB8, VAV2, and WASL The target proteins were knockdown by transfecting shROCK1, shVAV2, and shWASL in both TW02 and TW06 cells. (a) Cell migration and (b) wound-healing assays were measured in TW02 and TW06 cells. (c) Wound-healing and (d) transwell cell migration assays were performed after the TW02 cells were cotransfected with miRNA vector or miR-148a; and cDNA vector control or target gene expressing clones (ITGA11, ITGB8, VAV2, and WASL) as indicicated. The *p* values between 2 groups are indicated. (e) IHC analysis was performed to determine the expression levels of endogenous ROCK1, VAV2, and WASL in 21 paired NPC tissues. Only 6 paired NPC tissues are shown. (f) The correlation between miR148a RNA expression and the protein expression of 3 target genes in 5 paired NPC tissues were indicated. Protein expression fold change in each NPC tumor was normalized with the actin and non-tumor (T/N) (left panel). The expression fold change of miR-148a (T/N) quantitated using qRT-PCR and the fold change of the endogenous protein expression of target genes (T/N) in A-E NPC tissues were listed in a table (right panel). (g) The correlation of relative miR-148a levels in T/N (X-axis) and the endogenous protein levels of ROCK1, VAV2, and WASL in T/N (Y-axis) in 5 paired NPC tissues were indicated. (r: correlation coefficient)

The integrin signaling cascade, which regulates cell motility and migration, is frequently activated in cancer cells [26]. To investigate whether miR-148a inhibits migration via the targeting integrin pathway, we performed genes loss- and gain-of-function experiments on miR-148a target genes in the presence or absence of miR-148a in NPC cells. The knockdown of endogenous VAV2, ROCK1, and WASL alone triggered by individual shRNAs reduced cell migration by approximately 50% (Fig. 5a) as well as wound-healing ability (Fig. 5b) in TW02 cells, suggesting that these genes are involved in promoting cell migration. Next, we transfected miR-148a to knockdown its collective downstream target genes, and concurrently reintroduced an individual target gene sequentially to counteract the repressive effect of miR-148a in NPC cells. After 24 h of post-transfection, we performed transwell and wound-healing assays. The results showed that the cotransfection of miR-148a with each target gene (ITGA11, ITGB8, VAV2, and WASL) could promote cell migration (by approximately 20%) and wound-healing abilities (Figs. 5c-5d). These results revealed that, despite the expression of exogenous miR-148a, which inhibited the expression of hundreds of target genes, the reintroduction of an miR-148a target gene could restore the cell migration ability, suggesting that ITGA11, ITGB8, VAV2, and WASL are specific targets of miR-148a.

To test whether the endogenous miR-148a-target genes were overexpressed in 21 paired NPC tissues, we performed immunohistochemistry (IHC) to compare the expressions of ROCK1, VAV2, and WASL in paired NPC tissue array specimens (Pantomics). We graded each sample according to the IHC staining intensity (I) and the percentage of positive cells (P). The data showed that the expression levels of these 3 target genes in NPC tumors were typically higher than those of their adjacent normal tissues [average final score (T : N), ROCK1 (7.10 : 4.14), VAV2 (5.81 : 3.33), WASL (7.52 : 5.05)] (Fig. 5e) (Supplementary Table 6).

We subsequently wanted to test whether ROCK1, VAV2, and WASL protein expressions are inversely correlated with miR-148a levels in NPC tissues. We detected and compared the miR-148a levels as well as the protein levels of miR-148a target genes in the NPC paired samples. Because of the scarcity and limited size (2 to 3mm) of the NPC tissues, we selected only 5 of 15 paired NPC tissues. We previously conducted qRT-PCR by using these 5 NPC samples to analyze miR-148a expression levels in this study (Fig. 1c). The protein lysates of the same paired NPC samples were subjected to western blot analysis (ROCK1, VAV2, WASL, and Actin). First, the expression of miR-148a was downregulated to 0.02- to 0.37-fold in NPC tumors compared with the adjacent normal tissues (Fig. 5f, lower panel). Second, the protein expression (cut off>1.3-fold) of ROCK1, WASL, and VAV2 was upregulated in 3, 4 and 1 of the 5 NPC tumors, respectively (Fig. 5f). We observed a moderate negative correlation between miR-148a levels with ROCK1 (r = -0.52) and those with WASL (r = -0.45). However, we detected no such correlation between miR-148a levels and VAV2 because of the small sample size of the NPC tissues (Fig. 5g). Overall, these data showed that the low expression may correlate with the upregulation of the integrin signaling target genes ROCK1 and WASL in NPC tumors.

## DISCUSSION

In recent years, numerous reports have shown the critical role of epigenetic modifications in human cancers. A combination of epigenetic and genetic abnormalities can result in dysregulated gene expression and function, leading to tumors [27]. Altered epigenomic pattern such as the abnormal methylation of CpG islands at the gene promoter regions is one of the most common epigenetic alterations in cancer, affecting both coding and non-coding genes. Increasing evidence has shown that miRNAs are also epigenetically regulated. The aberrant DNA methylation of the miRNA upstream DNA sequence plays a significant role in cancer progression [27, 28]. The overexpression of DNMTs in NPC leads to abnormal hypermethylation and the inactivation of cellular genes [2]. We found that the inactivation of the non-coding gene miR-148a caused by DNA methylation within the DMR (-155 to +186) in NPC contributes to an increase in cell migration and promotes tumorigenesis. Our results showed that, for the first time, transcription factor USF1 activates the transcription of miR-148a; however, the methylated USF1 binding site (+131 to +137) interferes with the binding of USF1. Thus, the aberrant methylation of DMR (-155 to +186) suppresses miR-148a expression in NPC tissue.

Researchers have investigated the role of miR-148a in numerous cancers. The low expression levels of miR-148a have been detected, and are associated with an increase in tumor size and cancer metastasis [16, 18, 29, 30], implying that miR-148a can serve as a tumor suppressor miRNA by negatively regulating cellular oncogenes. Further studies have shown that the inactivation of miR-148a has led to the overproduction of oncogenic ROCK1, a kinase that promotes cell migration and invasion in gastric cancer[31]; Bcl-2, an antiapoptotic protein that increases cell survival in colorectal cancer [21]; and WNT10B, a signaling protein that stimulates β-catenin activation and cancer-associated fibroblast cell motility [8]. We also confirmed that at least 3 dysregulated oncogenic targets of miR-148a in NPC are genes that participate in the integrin-signaling pathway. The re-expression of miR-148a in NPC cells downregulated the protein expressions of ROCK1, VAV2, and WASL and reduced the cell migration ability, indicating that they are the bona fide targets of miR-148a.

Because miR-148a targets multiple integrin-pathway molecules, miR-148a inactivation may cause significant and additive effects in the upregulation of integrin signaling. Integrin-activated signaling pathways are crucial for cell growth, differentiation, and survival, especially cell invasion and metastasis [26, 32]. Each functional integrin molecule consists of 2 subunits: α and β. There are 18 and 8 subunits, respectively; different integrin combinations are responsible for binding to diverse extracellular matrix (ECM) ligands [33]. For example, binding to ECM fibronectin, integrins (α5/β1 and α5/β3) can promote cell migration [34]. Integrin clustering activates focal adhesion kinase (FAK) and SRC-family kinases (SFKs). This results in the activation of small GTPase in the Rho family [35] and downstream effectors such as (1) VAV, a guanine nucleotide exchange factor (GEF) for Rho GTPases (including cdc42, Rac, and RhoA); (2) WASL, a cdc42 effector; and (3) ROCK1, a downstream effector of RhoA. All of these effectors regulate actin polymerization, which is necessary for cell migration [36]. In addition, ITGA11 has been reported to be correlated with lymph node metastasis, and serves as a potential marker for the diagnosis of non-small cell lung cancer [37]. Furthermore, ITGB8 is an essential regulator of angiogenesis and tumor invasiveness in glioblastoma [38]. Previous studies have shown that VAV2 overexpression has resulted in increased invasion in oral squamous cell carcinoma and head and neck squamous cell carcinoma [39] [40]. WASL, a key regulator of cell migration, actin polymerization, and invadopodia induction [41], is highly expressed in hepatocellular carcinoma, and is considered a prognostic factor for overall survival [42]. ROCK1 plays a crucial role in promoting epithelial-to-mesenchymal transition in lung cancer [43] as well as invasion in gastric cancer [31]. Thus, the upregulation of the integrin downstream effector molecules appears to be common in different types of cancer, and is associated with tumor progression. In our study, we identified a novel link between epigenetically silenced miR-148a and integrin signaling activation in NPC. We showed that miR-148a could suppress ITGA11, ITGB8, VAV2, ROCK1, and WASL translation by binding to their 3′-UTR regions. Both ROCK1 and WASL have 2 miR-148a target sites on their 3′UTR. This may explain the reason the inhibitory effects of miR-148a toward ROCK1 and WASL are comparatively more significant compared with that toward VAV2, of which only one miR-148a target site exists. Consistent with this explanation, the inactivation of miR-148a in NPC tumors may result in a significant upregulation of ROCK1 and WASL.

**Figure 6:**
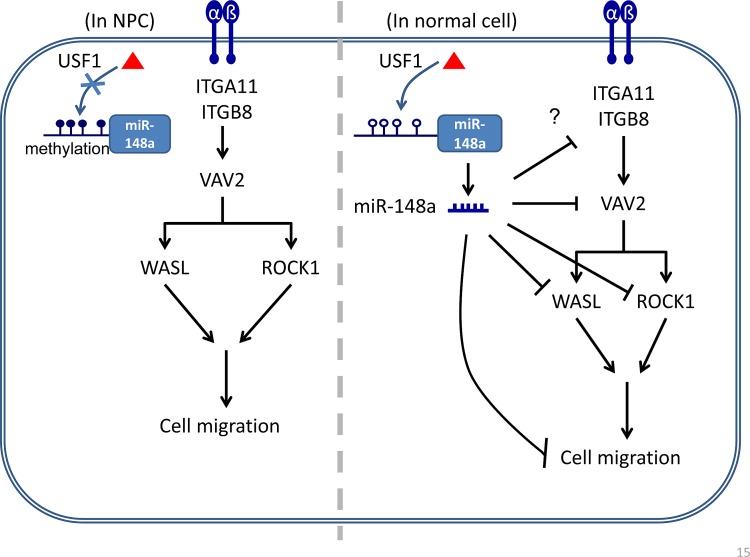
Schematic model of the silencing of miR-148a through aberrant hypermethylation enhances cell migration in NPC In normal cells, hypomethylated miR-148a promoter allows the binding of transcription activator USF1, resulting in the activation of miR-148a expression. Mature miR-148a represses the protein expression of the integrin pathway downstream targets such as ITGB8, VAV2, ROCK1 and WASL, and thereby inhibits cell migration. Conversely, in NPC cells, hypermehtylated miR-148a promoter prevents the binding of USF1 and causes silencing of miR-148a. In the absence of miR-148a, NPC cells overexpress oncogenic integrin pathway targets and, in turn, trigger cell migration.

In summary, to the best of our knowledge, silencing miR-148a through DNA methylation may have a substantial effect on gene expression. This is the first report to demonstrate that miR-148a can be activated by transcription factor USF1 by binding to the CG-containing a DNA consensus sequence, with its simultaneous silencing caused by DNA methylation. Investigating whether other gene promoters also depend on USF1 activation and whether they are also prone to silencing because of DNA methylation is warranted. Our findings revealed that miR-148a blocks the transcription of at least 3 downstream targets in the integrin pathway: ROCK1, VAV2, and WASL. Although miR-148a inhibits the protein expression of numerous target mRNAs, the contribution of each target is relatively small; a miRNA may typically inhibit approximately 20% of its target protein translation. However, the additive effect is significant when miR-148a targets multiple cellular effectors in the same pathway. We propose a model in which USF1 binding activates the promoter of miR-148a in normal tissue. The expression of miR-148a blocks the expression of the integrin pathway effectors ROCK1, VAV2 and WASL, thereby suppressing integrin signaling to inhibit cell migration (Fig. 6, right panel). Conversely, in NPC tumors, the miR-148a promoter is aberrantly hypermethylated, thus preventing the binding of transcription factor USF1, and resulting in a low expression of miR-148a. Unable to produce adequate miR-148a as a negative regulator of integrin pathway effectors, NPC tumors overexpress these effectors, hence promoting cell migration (Fig 6, left panel). Taken together, this report showed that miR-148a may be considered an antagonist of the integrin signaling pathway. Therefore, miR-148a may serve as a predictive and potential therapeutic biomarker for NPC and other cancers.

## MATERIALS AND METHODS

### Cell lines and NPC tissues

NPC cell lines (TW02 and TW06) and 293T [44] were cultured in 10%FBS DMEM medium. 293T cell was cultured in 10% FBS DMEM medium and 500μg/mL G418. NPC cell lines, HK1 [45] and C666.1 [46] were cultured in 10% FBS RPMI. Frozen NPC tumor and adjacent normal biopsies (<2mm) were collected from Chang Gung Memorial Hospital (CGMH; Taiwan) by Dr. K. P. Chang. This study was reviewed and approved by the IRB and ethics committee of CGMH (IRB: 97-1226A3).

### Bisulfite sequencing

Bisulfite-converted genomic DNA (100ng) (Zymo Research, Irvine, CA) was amplified by using bisulfite sequencing primers that were listed on Supplementary Table 5. The PCR products were cloned and 5~8 individual clones were sequenced.

### miRNA expression clones construction

The precursor miR-148a containing upstream and downstream 100bp flanking sequences were amplified using NP69 [22] genomic DNA as template. Sequences of primers are listed in Supplementary Table 5. PCR products were cloned into TA vector (Yeastern, Taiwan) and were further subcloned into expression vector pcDNA6.2-GW/EmGFP-miR using KpnI and NotI cloning sites (Invitrogen, modified by Dr. Chen, Hua-Chien).

### DNA transfection

Two microgram miRNA expression clones and vector control were transiently transfected into TW02 and TW06 cells (2×10^5^), respectively, 48hrs in 6-well plate by Lipofectamine (Invitrogen, Carlsbad, CA, USA). Fifty pmol of miRIDIAN hairpin inhibitor (miR-148a inhibitor, MI0000253, Dharmacon) or negative control (IN-001005-01-05, Dharmacon) were co-transfected with either vector or miR-148a as required. Cells were harvested after 48hrs transfection, and RNA was extracted to assess the miRNA expression level by stem-loop qRT-PCR [47].

### RNA extraction, reverse transcription (RT) and quantitative RT-PCR

Total RNA was extracted by using Trizol reagent (Invitrogen, Carlsbad, CA, USA) according to the manufacturer's protocol. Extracted RNA (10μg) was treated with RNase-free DNase RQ1 (10U; Promega) to remove trace amount of genomic DNA and then followed by another Trizol extraction. For miRNA, reverse transcription and PCR was carried out by using Superscript III transcriptase (Invitrogen) and was conducted as previously described [47] [12]. The corresponding primers were listed in Supplementary Table 5. For cellular mRNA, reverse transcription was carried out by using ImProm-II^TM^ Reverse Transcription system (Promega) according to the manufacturer's protocol.

### Immunoblotting

20 μg of protein lysate was analyzed by immunoblotting by using antibodies against VAV2 (sc-10803; Santa Cruz), WASL (H00008976-M04; Abnova), ROCK1 (NB110-57465; Novus) and Actin (MDBio, Taiwan) and appropriate horseradish peroxidase (HRP)-conjugated secondary antibody at 1:10000 dilution. The immunoblot was detected by the enhanced chemiluminescence system (Millipore), and exposed to X-ray film.

### 3′UTR construction and Luciferase reporter assay

3′UTR sequences of miR-148a target genes containing upstream and downstream 100bp flanking sequences of the putative miR148a target sites were amplified from 293T genomic DNA by PCR and cloned into the pMIR-Report-Vector (Ambion, USA). Primers used for PCR were listed on Table 4. 3′UTR luciferase reporter assays were performed by calcium phosphate transient transfection of 293T cells (2×10^5^) with 20ng of pMIR-reporter- 3′UTR, and 200ng of the vector control or miR-148a expression clone and 10ng pCMV-Renilla (internal control). Dual Luciferase Assay (Promega, USA) was used to measure the luciferase and renilla activities. All the luciferase values were normalized to that of the Renilla values and the ratio of firefly/ renilla was presented.

### Transwell assay

TW02 and TW06 cells (2×10^5^) were transient transfected with or without miR-148a and pCMV-3tag based target genes. Post transfection 48hr, 1.5×10^5^ cells were resuspended in 300μl serum free medium and seeded to the transwell of uncoated polycarbonate membranes with 8.0μm pores (BD Bioscience) with the bottom supplemented with 800μl complete medium. After 20hr incubation, cell migrated to the other side of transwell were stained with 0.005% crystal violet. Ten *photographs were taken* randomly and the cell number was counted.

### shRNA transfection

The NPC cell lines TW02 and TW06 (2×10^5^) were plated onto 6-well culture plates the day before transfection. shRNAs plasmids of each target genes and vector control (2μg), purchased from the National RNAi Core Facility, Academia Sinica, Taiwan, was transiently transfected with Lipofectamine reagent (Invitrogen).

### Wound-healing assay

NPC cells were cultured in 24-well plates at 5× 10^4^ cells/well as monolayers. After post-transfection 48hr, the cells were wounded in three lines across the well with a 10μl pipette tip. The area of wounds was then captured at 0 and 8hr by microscope. The images were analyzed by ImageJ software. The wound healing assay was calculated as the fold change of the remaining gap area compared with the area of the initial wound.

### Electrophoretic mobility shift assay (EMSA)

EMSA assay was performed as described previously [30].

### Immunohistochemistry (IHC)

Immunohistochemistry was performed by Chang Gung University Pathology Core Center using NPC tissue array (NPC961; Pantomics, Richmond, CA). Antibodies for ROCK1, VAV2 and WASL were used by immunoblotting assay. Brief description of protocol is reported according to the previously described procedures [48] [49]. The staining intensity was graded as 0~4 to indicate undetectable, weak, moderate, strong, and extremely strong staining, respectively. The staining area was graded as 0~4 to indicate the percentage of cells that showed negative, <25%, 25~50%, 50~75%, and >75%, respectively. The score of each sample was multiplied by the grading of intensity and staining area.

All the data presented in quantitation RT-PCR, transwell assays, DNA transfection, luciferase assays and wound-healing assays were the results from three independent, duplicate experiments, the results are shown as mean±SD. (*p<0.05; **p<0.01; ***p<0.001*)*.

## SUPPLEMENTARY FIGURES AND TABLES




