# Supplementary material for: Silencing of miRNA-148a by hypermethylation activates the integrin-mediated signaling pathway in nasopharyngeal carcinoma
Source: Oncotarget. 2014 Jul 31;5(17):7610–24. doi: 10.18632/oncotarget.2282 (PMC4202148; doi:10.18632/oncotarget.2282)
Supplement: Supplementary file 1 [file oncotarget-05-7610-s001.docx]

**Supplementary figures and tables**

**Table 1.** Significantly upregulated microRNAs in C666.1 after the treatment of DNA demethylation agent 5’aza (10μM). (Agilent miRNA microarray)

| **Name** | **C666.1** | **C666.1+5’aza** | **Fold change**  **(+/- 5’aza)** |
| --- | --- | --- | --- |
| hsa-miR-9 | 8.55 | 28.86 | 3.37 |
| hsa-miR-21 | 4211.36 | 6874.22 | 1.63 |
| hsa-miR-132 | 36.42 | 104.32 | 2.86 |
| hsa-miR-148a | 10.31 | 67.42 | 6.54 |
| hsa-miR-449a | 329.03 | 1006.84 | 3.06 |
| hsa-miR-1308 | 28.97 | 114.71 | 3.96 |

**Table 2.** The **i**ntersected 316 genes were analyzed by MetaCore (Pathway Map analysis) and the top five statistically significant pathways are listed.

| # | Pathway Maps: statistically significant maps | pValue | Ratio |
| --- | --- | --- | --- |
|  |  |  | hit/ total |
| 1 | Cell adhesion: Chemokines and adhesion | 4.839E-07 | 10/ 100 |
| 2 | Cytoskeleton remodeling: Cytoskeleton remodeling | 5.321E-06 | 9/ 102 |
| 3 | Cytoskeleton remodeling: TGF, WNT and cytoskeletal remodeling | 1.068E-05 | 9/ 111 |
| 4 | Cell adhesion: Integrin-mediated cell adhesion and migration | 3.358E-04 | 5/ 48 |
| 5 | Cytoskeleton remodeling: Fibronectin-binding integrins in cell motility | 5.973E-04 | 4/ 31 |

**Table 3.** Intersected genes between (i) miR-148a predicted targets (TargetScan) and (ii) upregulated genes (T/N $\geq$ 1.3) in NPC tumors that are involved in cell migration.

| **Function** |  | **Gene** | **Fold change (T/N)** | **p-value** |
| --- | --- | --- | --- | --- |
| Migration, motion, adhesion |  | DLL4 | 1.30 | 0.0665 |
|  |  | TNXB | 1.30 | 0.0256 |
|  |  | FLOT2 | 1.30 | 0.4157 |
|  | * | ROCK1 | 1.34 | 0.0429 |
|  | * | VAV2 | 1.41 | 0.3661 |
|  | * | ITGB8 | 1.45 | 0.1579 |
|  |  | POU3F2 | 1.46 | 0.2159 |
|  |  | FEZ2 | 1.46 | 0.1270 |
|  |  | CSF1 | 1.47 | 0.0469 |
|  |  | GAP43 | 1.53 | 0.0637 |
|  |  | ADAM17 | 1.57 | 0.1623 |
|  | * | ITGA11 | 1.78 | 0.1192 |
|  | * | WASL | 2.11 | 0.0319 |
|  |  | COL6A3 | 2.39 | 0.3516 |
|  |  | ROBO1 | 3.70 | 0.0030 |

* Genes that are involved in integrin signaling pathway.

**Table 4.** Fold change of mRNA expression level (qRT-PCR) of miR-148a target genes in the miR-148a overexpressing NPC cells versus control.

| **Gene** | **Fold change**  **(+/- miR-148a)** | **p-value** |
| --- | --- | --- |
| FEZ2 | 0.7266 | 5.75E-07 |
| ADAM17 | 0.7468 | 6.43E-06 |
| *WASL | 0.7367 | 2.83E-07 |
| *ITGA11 | 0.7855 | 4.16E-06 |
| *ROCK1 | 0.7951 | 1.63E-02 |
| FLOT2 | 0.8012 | 2.30E-03 |
| ROBO1 | 0.8209 | 3.17E-04 |
| *ITGB8 | 0.8544 | 7.70E-02 |
| TNXB | 0.8853 | 2.21E-04 |
| POU3F2 | 0.9542 | 3.00E-01 |
| GAP43 | 0.9754 | 6.95E-01 |
| *VAV2 | 1.0796 | 1.15E-01 |
| COL6A3 | 1.1222 | 5.25E-01 |

* Genes that are involved in integrin signaling pathway.

**Table 5. Primer sequences.** (a) Primers for miR-148a expression clones construction, miR-148a-specific stem-loop RT reaction and miR-148a qRT-PCR. (b) Primers for target genes’ 3’UTR clone construction.

**(a)**

| miR-148a expression clone | F'-NotI: TTGGCGGCCGCCTTTTAACGAGTTATTCTTCTTTG  **R'-KpnI:** CCC**GGTACC**GCCTTGCCCCTCCCCCAAGGG |
| --- | --- |
| miR-148a-specific stem-loop RT primer | 5’-CTCAACTGGTGTCGTGGAGTCGGCAATTCAGTTGAGACAAAGTT-3’ |
| miR-148a-specific forward primer for qPCR | 5’-CGGCGGTCAGTGCACTACAGAA-3’ |

**(b)**

| Target gene | 3’UTR clones |
| --- | --- |
| ITGA11 | F’-SpeI: ACTAGT AAGCATGTGGATGACACAATCC  R’-HindIII: AAGCTT ATCACTGGTCATACCACTTGTC |
| ITGA11(mut) | F’: TGGAAGTACAGTCATAAAGCACGTGCAAGGACTCCC  R’: TTTATGACTGTACTTCCAGAGGGGACCCCTCAACAG |
| ITGB8-1 | F’-SpeI: ACTAGTCCTCTGAAGAGCACTGATTACAC  R’-HindIII: AAGCTTAGCTGTTCTTGTCTATTCAACCACC |
| ITGB8-1(mut) | F’:ATCCCGTACAGTGACATGTGAGGAAAAAAATAATC  R’:ATGTCACTGTACGGGATTACTGTACCCTTGTAGTG |
| ITGB8-2 | F’-SpeI: ACTAGTAATGTGCCATACATACACTACAAC  R’-HindIII: AAGCTTAGTCCTCCAGAAAAGTCTATGACTT |
| ITGB8-2(mut) | F’:ACCATGTACAGTCAGTGCACACGTATTTATAAACA  R’:CACTGACTGTACATGGTGTCCCCAGAGAAATAAAA |
| VAV2 | F’-SpeI: ACTAGTGATCACGAAGGACACTGAGAAA  R’-HindIII: AAGCTTATGTTCACAGCTAATCTCAGG |
| VAV2 (mut) | F’: GTTTTGTACAGTCACTAAGGCAGGAGGGTTGGAGG R’: TAGTGACTGTACAAAACCAAACCAAACACAAATCT |
| WASL- | F’-SpeI: ACTAGTCTGTGGAGTTCAGTCCAGGCA  R’-HindIII: AAGCTTTTGCCACCAGTGCAACATCCT |
| WASL (mut1) | F’: CATCCGTACAGTTGAGCAGCACTATACCTGTGCGTTG  R’: GCTCAACTGTACGGATGGTAGAGGACTGAAACATGCA |
| WASL (mut2) | F’: GATGTGTACAGTGTGGCAAAAGCTTAATAAAGGATCT R’: GCCACACTGTACACATCCTATTCACTATACATTTCA |
| ROCK1-1 | F’-SpeI: ACTAGTCTAGAGACATTAGGAAACTAACTG  R’-HindIII: AAGCTTCTATACTTACTCATGGCAGT |
| ROCK1-1 (mut) | F’: ATGTAGTACAGTTTTGAAATGTAAATTATTCTTAG  R’: TCAAAACTGTACTACATTTCTTATATGTTTGTTAA |
| ROCK1-2 | F’-SpeI: ACTAGTGTCCAGTAACAAGAGTTGATTC  R’-HindIII: AAGCTTCCATGAGAAAACACATTGCAGT |
| ROCK1-2 (mut) | F’: AAGCCTACAGTCTGGATATGTAATAATAAACATAT  R’: ATCCAGACTGTAGGCTTTCAATACCACTTGAAACA |

**(c)**

| Region | MiR-148a promoter |
| --- | --- |
| -1741~+45 | F’-MluI-ACGCGTATTTCGCACTCTGCCCTGCAGCAGC  R’-BglII-GACAGATCTCATACTCAGAGTCGGAGTGTCTCAG |
| -870~+45 | F’-MluI -CAGACGCGTTGCACGCGCGCAACAGCTGTTCAG  R’-BglII -GACAGATCTCATACTCAGAGTCGGAGTGTCTCAG |
| -1741~+218 | F’-MluI -ACGCGTATTTCGCACTCTGCCCTGCAGCAGC  R’-BglII -AGATCTGCTTCAAGGGAATTGGTCAAGTTC |
| -870~+218 | F’-MluI -CAGACGCGTTGCACGCGCGCAACAGCTGTTCAG  R’-BglII -AGATCTGCTTCAAGGGAATTGGTCAAGTTC |
| -155~+218 | F’-MluI-ACGCGTCAGTAACTTTCCATAATTAATGACCCG  R’-BglII -AGATCTGCTTCAAGGGAATTGGTCAAGTTC |

**(d)**

| cDNA clones | Constructed in pCMV-3Tag vector |
| --- | --- |
| ITGA11 | F'-HindIII-AAGCTTATGGACCTGCCCAGGGGCCTG  R'-XhoI-CTCGAGCTCCAGCACTTTGGGGGTGG |
| ITGB8 | F'-BamHI-GGATCCATGTGCGGCTCGGCCCTGGCTT  R'-XhoI-CTCGAGGAAGTTGCACCTGAAAGTTTC |
| VAV2 | F'-HindIII-AAGCTTATGGAGCAGTGGCGGCAGTG  R'-XhoI-CTCGAGCTGGATGCCCTCCTCTTCTAC |
| WASL | F’-HindIII-AAGCTTATGAGCTCCGTCCAGCAGCAGCC  R’-XhoI-CTCGAGGTCTTCCCACTCATCATCATCCTC |
| GATA1 | F’-SacI-AGAGAGCTCATGGAGTTCCCTGGCCTGGGG  R’-XhoI-GTGCTCGAGTGAGCTGAGCGGAGCCAC |
| GATA2 | F’-NotI-CCGGCGGCCGCATGGAGGTGGCGCCCGAGCAG  R’-XhoI-TGTCTCGAGGCCCATGGCGGTCACCATGCTG |
| USF1 | F’-BamHI-CGGATCCATGAAGGGGCAGCAGAAAACAGC  R’-XhoI-CCTCGAGGTTGCTGTCATTCTTGATGACGA |

**(e)**

| Mutated site | MiR-148a promoter (DMR) |
| --- | --- |
| CDP-CR (mut) | F’-GACCAATTCCATTATTCTAGAAATCCTGAACTAAATTG  R’-GGATTTCTAGAATAATGGAATTGGTCATTAATTATG |
| GATA1/2 (mut) | F’-GTGGTCTCCCTCTAGACCAGTGCTCCAGACGC  R’-CACTGGTCTAGAGGGAGACCACAGCCTCTAGA |
| USF1 (mut) | F’-GGCCATTCTAGAAATCCTTTCCCTTGGGGG R’-AAAGGATTTCTAGAATGGCCACCAGGGCCA |

**(f)**

| probe | EMSA probes |
| --- | --- |
| Wild type (biotin) | F’-Biotin-GTGGCCACGTCGCTGC  R’-Biotin- GCAGCGACGTGGCCAC |
| Methylated (biotin) | F’-Biotin-GTGGCCA*CGT*CGCTGC  R’-Biotin-GCAG*CGA*CGTGGCCAC |
| Wild type (cold) | F’- GTGGCCACGTCGCTGC  R’- GCAGCGACGTGGCCAC |
| Mutant (cold) | F’--GTGGCCATTCTAGAAA  R’- TTTCTAGAATGGCCAC |

**Table 6. Endogenous expression of miR-148a targets in 21 paired NPC tissues.**

| **NPC** | **ROCK1** | | **VAV2** | | **WASL** | |
| --- | --- | --- | --- | --- | --- | --- |
|  | **T** | **N** | **T** | **N** | **T** | **N** |
| **A2** | 4 | 2 | 16 | 1 | 9 | 1 |
| **A3** | 9 | 4 | 6 | 2 | 12 | 2 |
| **A4** | 6 | 4 | 1 | 1 | 4 | 1 |
| **A6** | 9 | 1 | 4 | 2 | 6 | 4 |
| **A7** | 16 | 4 | 12 | 4 | 12 | 4 |
| **A8** | 9 | 4 | 3 | 6 | 9 | 4 |
| **A9** | 1 | 12 | 1 | 2 | 6 | 6 |
| **A10** | 1 | 4 | 4 | 6 | 16 | 2 |
| **A11** | 9 | 6 | 12 | 9 | 2 | 6 |
| **A12** | 6 | 1 | 2 | 2 | 3 | 12 |
| **C2** | 1 | 1 | 2 | 4 | 2 | 9 |
| **C3** | 6 | 2 | 6 | 6 | 12 | 4 |
| **C4** | 6 | 1 | 9 | 2 | 9 | 1 |
| **C5** | 12 | 1 | 9 | 1 | 3 | 1 |
| **C6** | 1 | 9 | 1 | 1 | 1 | 2 |
| **C7** | 6 | 3 | 1 | 1 | 6 | 2 |
| **C10** | 16 | 6 | 12 | 4 | 8 | 9 |
| **E1** | 3 | 6 | 4 | 6 | 4 | 2 |
| **E3** | 4 | 6 | 4 | 4 | 6 | 16 |
| **E6** | 12 | 6 | 4 | 4 | 12 | 9 |
| **E7** | 12 | 4 | 9 | 2 | 16 | 9 |
| **Average** | **7.10** | **4.14** | **5.81** | **3.33** | **7.52** | **5.05** |
| **T-test** | **0.0177** | | **0.0281** | | **0.0728** | |
| **T>N** | 15 | | 9 | | 14 | |
| **N>T** | 5 | | 5 | | 6 | |
| **N=T** | 1 | | 7 | | 1 | |
| **total** | 21 | | 21 | | 21 | |

Final score (FS) was obtained by multiplying the IHC staining intensity (I: 1-4 points) and the percentage of positive cells (P: 1-4 points). FS=I$\times$P, maximum final score=16.
