# Supplementary material for: Silencing of miRNA-148a by hypermethylation activates the integrin-mediated signaling pathway in nasopharyngeal carcinoma
Source: Oncotarget. 2014 Jul 31;5(17):7610–24. doi: 10.18632/oncotarget.2282 (PMC4202148; doi:10.18632/oncotarget.2282)
Supplement: Supplementary file 2 [file oncotarget-05-7610-s002.pdf]

Silencing of miRNA-148a by hypermethylation activates the integrin-mediated signaling pathway in nasopharyngeal carcinoma

Supplementary Material

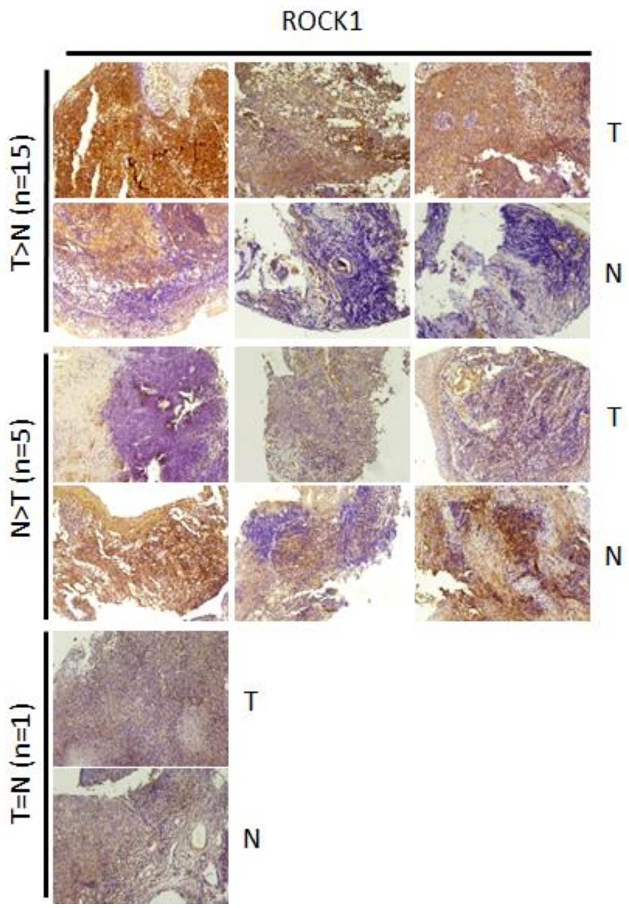

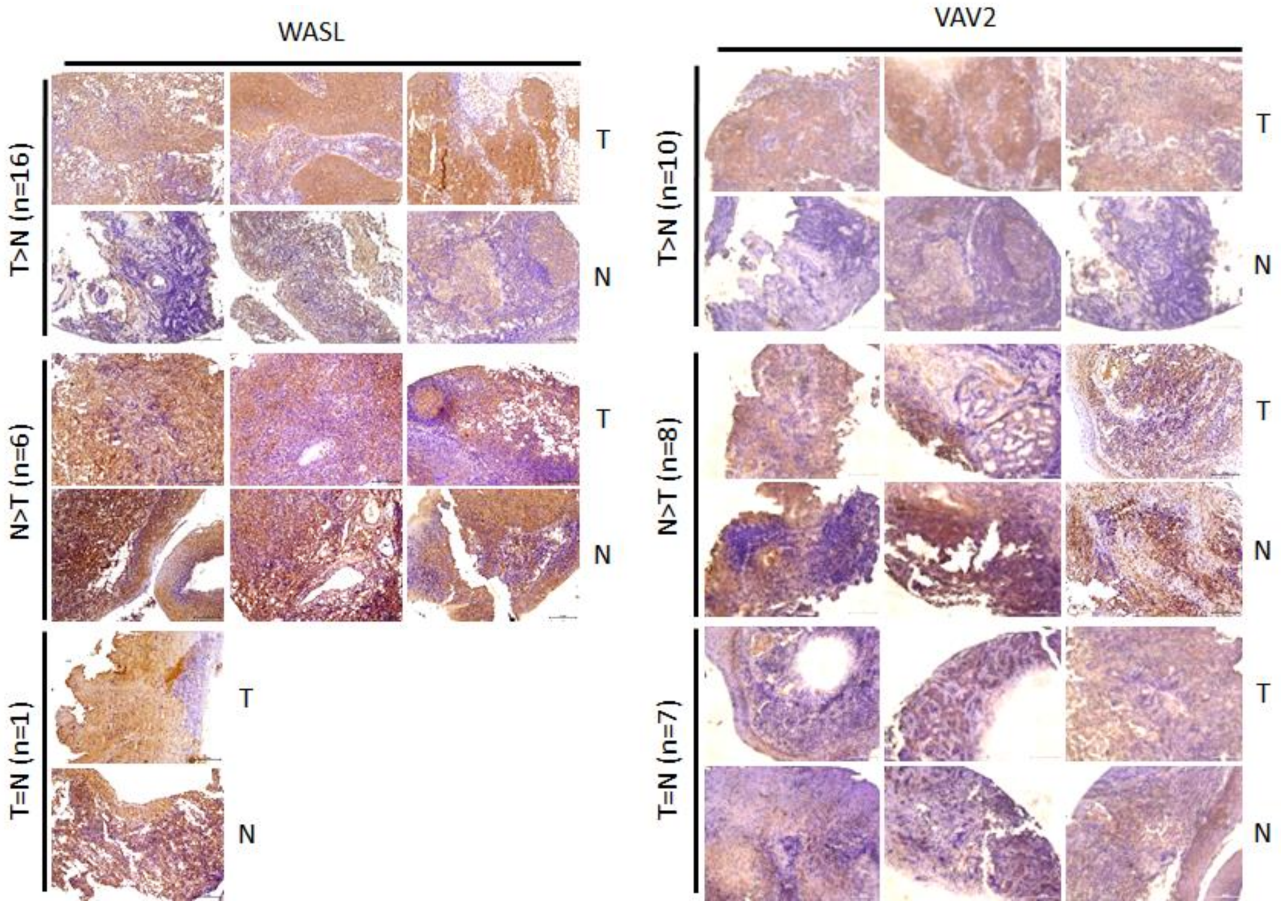

Supplementary Figure 1: Endogenous expression of miR-148a targets in 21 paired NPC tissues.
